# Repeated Aconitine Treatment Induced the Remodeling of Mitochondrial Function *via* AMPK–OPA1–ATP5A1 Pathway

**DOI:** 10.3389/fphar.2021.646121

**Published:** 2021-06-10

**Authors:** Li-Zhen Qiu, Wei Zhou, Lan-Xin Yue, Yi-Hao Wang, Fei-Ran Hao, Peng-Yan Li, Yue Gao

**Affiliations:** ^1^ State Key Laboratory of Component-based Chinese Medicine, Tianjin University of Traditional Chinese Medicine, Tianjin, China; ^2^ Department of Pharmaceutical Sciences, Beijing Institute of Radiation Medicine, Beijing, China; ^3^ The Fifth Medical Center, General Hospital of PLA, Beijing, China

**Keywords:** aconitine, mitochondrial homeostasis, mitochondrial fusion, OPA1, ATP5A1

## Abstract

Aconitine is attracting increasing attention for its unique positive inotropic effect on the cardiovascular system, but underlying molecular mechanisms are still not fully understood. The cardiotonic effect always requires abundant energy supplement, which is mainly related to mitochondrial function. And OPA1 has been documented to play a critical role in mitochondrial morphology and energy metabolism in cardiomyocytes. Hence, this study was designed to investigate the potential role of OPA1-mediated regulation of energy metabolism in the positive inotropic effect caused by repeated aconitine treatment and the possible mechanism involved. Our results showed that repeated treatment with low-doses (0–10 μM) of aconitine for 7 days did not induce detectable cytotoxicity and enhanced myocardial contraction in Neonatal Rat Ventricular Myocytes (NRVMs). Also, we first identified that no more than 5 μM of aconitine triggered an obvious perturbation of mitochondrial homeostasis in cardiomyocytes by accelerating mitochondrial fusion, biogenesis, and Parkin-mediated mitophagy, followed by the increase in mitochondrial function and the cellular ATP content, both of which were identified to be related to the upregulation of ATP synthase α-subunit (ATP5A1). Besides, with compound C (CC), an inhibitor of AMPK, could reverse aconitine-increased the content of phosphor-AMPK, OPA1, and ATP5A1, and the following mitochondrial function. In conclusion, this study first demonstrated that repeated aconitine treatment could cause the remodeling of mitochondrial function *via* the AMPK–OPA1–ATP5A1 pathway and provide a possible explanation for the energy metabolism associated with cardiotonic effect induced by medicinal plants containing aconitine.

## Introduction

Aconitine is one of the main bioactive ingredients in *Aconitum L*. Apart from the toxicity, this aconite alkaloid is attracting ever-growing attention for its potential pharmacological effects on the cardiovascular system, such as anti-shock, anti-inflammatory, and myocardial protection effects induced at low doses (Xie and Peng, 2017). Notably, since the inotropic effects of aconitine were detected in the isolated muscles, some scholars had focused on the potential cardiotonic effect of low-dose aconitine. For instance, 0.01 mg/kg of acontine has been identified to significantly improve the cardiac function of rats by enhancing left ventricular systolic pressure and left ventricular end-diastolic pressure. Recent studies also found that low doses of aconitine could generate obvious cardiotonic effect in heart failure (Zhang and Wu 2001; Wen et al., 2012; Wang et al., 2019; Liu et al., 2020), especially combining with acupuncture treatment. Hence, Chan et al. and many Chinese experts on aconite shared a viewpoint that aconitine could generate a positive inotropic effect possibly by increasing the intracellular sodium content during the action potential (Chan, 2009), providing a novel pharmaceutical use of this compound.

The maintenance of the positive inotropic effect featuring enhanced cardiac contractility with high frequency always requires coordinated energy supply in cardiomyocytes. However, how to guarantee proper regulation of energy metabolism that fits for cardiotonic effects induced by aconitine in cardiac myocytes remains to be explored. As the major sites of energy synthesis in the heart, mitochondria are double-membraned subcellular organelles that consist of outer membranes, inner membranes, and soluble matrix surrounded by the inner membrane (Marin-Garcia and Akhmedov, 2016). The inner mitochondrial membrane folds inwardly to form highly organized invaginations known as cristae, which are studded with various protein complexes, such as respiratory chain supercomplexes, and participate in the generation of ATP (Upadhyay and Agarwal, 2020). Our latest work has shown that low dose of aconitine could improve the energy metabolism disorder by reducing CypD-mediated mPTP, resulting in the restoration of angiotensin II–induced myocardial mitochondrial dysfunction (Wang et al., 2021). Thus, a synchronized supply of ATP and/or energy metabolism in elevated contracted cardiomyocytes could be ascribed to aconitine-induced alterations in mitochondrial function.

Structurally, mitochondria are being dynamic homeostasis (mitochondrial turnover), that is, the principal regulator of mitochondrial morphology and function and is largely facilitated by mitochondrial fission, mitochondrial fusion, and mitophagy (Fuhrmann and Brune, 2017; Fu et al., 2019). Generally, mitochondrial biogenesis and fusion both optimize mitochondrial function and counteract the increasing demand of energy (Panchal and Tiwari, 2019). In recent, benzoylaconine, the main metabolite of aconitine, has been demonstrated to enhance the mitochondrial mass *via* promoting mitochondrial biogenesis, suggesting that aconitine might increase myocardial ATP content by exerting a crucial impact on mitochondrial turnover. However, the underlying mechanism is not fully elucidated, nor does the effect of aconitine on mitochondrial fusion.

OPA1, a dynamin-like 120 kDa protein that could promote the formation and modification of crista, mainly participates in the regulation of mitochondrial fusion and following ATP production (Baker et al., 2014). Besides, AMP-activated protein kinase (AMPK), acting as a key regulator of mitochondrial function and energy metabolism, has been found to improve mitochondrial quality control through increasing OPA1 expression in the myocardium (Zhang et al., 2019; Xin et al., 2020), implying that the potential role of AMPK/OPA1 signaling in mitochondrial fusion. But so far, there is no direct evidence of AMPK/OPA1 signaling-mediated mitochondrial fusion present in acontine-induced positive inotropic effect on the myocardium.

Hence, we hypothesized that repeated administration of low doses of aconitine could generate a positive inotropic effect on ventricular cardiomyocytes through AMPK/OPA1-mediated remodeling of mitochondrial function. Also, this study was designed to verify the hypothesis and explored the possible mechanism involved.

## Materials and Methods

### Study Animals

Neonatal Wistar rats (1 day) were purchased from SPF Laboratories (Beijing, China, Beijing Vital River, certificate No. SCXK 2016–0011). All animal experiments were approved by the Ethics Committee of the Beijing Institute of Radiation Medicine (Approval No. IACUC-DWZX-2020–771).

### Primary Neonatal Rat Ventricular Myocyte Culture

NRVMs were obtained from neonatal Wistar rat pups *via* serial enzymatic digestion. In brief, after cervical dislocation and disinfection, the heart tissues were immediately isolated and placed into the ice-cold phosphate buffer solution (PBS) (Gibco, Thermo Fisher Scientific, Waltham, MA, United States). After rinsing 4 times, the ventricular tissues were cut into less than 1 mm^3^ tissue block and digested in trypsin (0.0625%, Sigma-Aldrich, St. Louis, MO, United States) for 5 min at room temperature (RT), followed by 5 min collagenase II (1 mg/ml, Solarbio, Beijing, China) enzymolysis at 37°C. Then the supernatant fluid was filtered using a sterile 70 μm cell strainer and transferred into a centrifuge tube containing Dulbecco’s modified Eagle medium (DMEM) (Gibco) supplemented with 15% fetal bovine serum (FBS) (Gibco) and 1% penicillin/streptomycin (Hyclone, Logan, UT, United States). After further purification by eliminating fibroblasts, NRVMs were replanted into the 100 mm dishes (2×10^6^ cells/ml), combining with 5-BrdU to inhibit cardiac fibroblast growth for 48 h. 0.1 mM 5-BrdU (Sigma-Aldrich), and cultured in a humidified atmosphere at 37°C and 5% CO_2_.

### Drug Treatment and CCK-8 Assay

NRVMs were repeatedly treated with aconitine (purity ≥98%, ManSiTe, Chengdu, China) at a series of diluted concentrations (0, 2.5, 5, 10, 20, 40, and 80 μM, respectively) or dopamine (DA, 50 μM, Solarbio) for 7 days, and the drug was refreshed every 24 h. At the end of treatment, cell viability was detected by the Cell Counting Kit-8 assay (CCK-8, Dojindo, Tokyo, Japan) according to the manufacturer’s instruction. In brief, NRVMs were washed twice and cultured with DMEM medium containing 10% CCK-8 reagent for another 2 h, and then the absorbance was detected using a microplate reader at 450 nm (Multiskan MK3, Thermo Fisher Scientific). The cell viabilities were presented as the percentages of that of the control group.

### Quantification of Mitochondrial Superoxide

Mitochondrial superoxide was determined using the MitoSOX Red indicator (M36008, Thermo Fisher Scientific). NRVMs were seeded into a black 96-well plate (clear bottom with lid) and repeatedly exposed to aconitine (0, 2.5, 5, 10, 20, 40, and 80 μM, respectively) or DA (50 μM) for 7 days. After treatment, cells were washed with PBS buffer and then incubated with 5 μM MitoSOX Red for 10 min at 37°C. Next, fluorescence intensities of each group were detected with a multilabel microplate reader (Victor X5, PerkinElmer, Waltham, MA, United States), and results were presented as mean ± SD.

### Western Blotting

Western blotting was performed according to the previous description, and treated NRVMs were harvested and immediately lysed using a Minute™ Total Protein Extraction Kit (SD-001, Invent, Beijing, China) supplemented with protease/phosphatase inhibitor cocktail (P1265/P1260, Applygen, Beijing, China), according to the manufacturer’s instruction. The supernatants of total proteins were collected, and the concentrations were quantified *via* a BCA assay kit (P1510, Applygen). The denatured proteins were separated by SDS-PAGE gels and electrotransferred onto polyvinylidene fluoride (PVDF) membranes (Millipore, Billerica, MA, United States). After overnight blocking in 5% nonfat milk at 4°C, the membranes were incubated orderly with primary antibodies and corresponding secondary antibodies (detailed information for all indicated antibodies was listed in Supplementary Table S1). After rinsing three times with TBST, the protein blots were visualized with a chemiluminescence ECL Western blot system (Millipore) and an automatic exposure system (Image Quant LAS500, GE, Fairfield, CT, United States). The grayscale values of the bands were determined by ImageJ software; GAPDH or ß-actin were used as loading controls for total proteins.

### Transmission Electron Microscopy

At the end of treatment, NRVMs were harvested and were fixed in 100 mM phosphate buffer (PB) (pH = 7.2) containing 2% formaldehyde and 2.5% glutaraldehyde overnight at 4°C. Then, the samples were washed three times with 0.1 M PBS and postfixed using 1% osmium tetroxide for 4 h at RT, followed by dehydration in a series of ascending concentrations of ethanol solutions (50, 70, 80, and 100%). Subsequently, cells were subject to propylene oxide and embedded in epoxy resin. Afterward, the blocks were sliced into ultrathin sections, which were then double-stained with 2% uranyl acetate and 5% lead citrate and finally observed using a transmission electron microscope (H-7650, HITACHI, Japan). The area and the number of mitochondria were analyzed by ImageJ software.

### Confocal Microscopy and Quantification of Mitochondrial Fusion

NRVMs suspensions (1×10^5^ cells/ml) were seeded in confocal dishes and exposed to aconitine (0 or 5 μM) with or without CC co-treatment for 7 days. At the end of treatment, cells were incubated with DMEM containing 200 nM MitoTracker Red CMXRos probe (M7512, Thermo Fisher Scientific) for 30 min at 37°C. After two rinses with PBS, cells were fixed with 4% formaldehyde for 10 min and blocked with blocking buffer (P0102, Beyotime, Beijing, China) for 1 h at RT. Next, the nuclei were counterstained with 4′,6-diamidino-2-phenylindole (5 μg/ml, DAPI, Sigma-Aldrich) for 7 min at RT. After 5 min wash (×3), the cells were visualized using a laser scanning confocal microscope (LSM 880, Carl Zeiss, Jena, Germany).

The obtained confocal images were subjected to the quantitative analysis of mitochondrial morphology using ImageJ software. In brief, the type of images was first converted into an 8-bit grayscale form. Next, the images’ background noise removal was carried out at a threshold value which might distinguish individual mitochondrial fragments in a single cell. Then the photographs were converted into binary images; the number of noncontiguous mitochondrial fragments and the area of these mitochondria were calculated by ImageJ’s particle counting subroutine. For each image, the number of mitochondria was normalized by the mitochondrial area and then × 1000 to gain the mitochondrial fragmentation index (MFI), which was proved as a verified index for mitochondrial fragmentation. For each group, 8–25 randomly selected cells were used to calculate the MFI values.

### Seahorse XF96 Respirometry

To assess the effects of aconitine on mitochondrial function, the oxygen consumption rate (OCR) and extracellular acidification rate (ECAR) were both analyzed using a Seahorse XF96 extracellular flux analyzer (Seahorse Bioscience, Agilent, TX, United States). Briefly, NRVMs (20,000 cells *per* well) were seeded in XFe96 cell culture microplates (101085-004, Agilent) and repeatedly treated with aconitine (5 μM) for 7 days, with or without CC co-treatment. Before assay, the cells were equilibrated in fresh XF DMEM base medium (103575-100, Agilent) containing 25 mM glucose (103577-100, Agilent), 1 mM sodium pyruvate (103578-100, Agilent), and 2 mM L-glutamine (103579-100, Agilent) for 1 h at 37°C without CO_2_. For the Mito Stress test, 2 μM oligomycin A (oligo), 2 μM carbonyl cyanide-p-trifluoromethoxy-phenylhydrazone (FCCP), and 0.5 μM rotenone/antimycin A (A/R) were sequentially injected into each well to perform consecutive OCR measurements. For ATP rate assay, only 2 μM oligo and 0.5 μM A/R were added successively to obtain ECAR value. OCR and ECAR data were used to assess mitochondrial respiratory capacity and ATP production rate according to the manufacturing instructions of Seahorse XF Mito Stress Test Kit (103015-100, Agilent) or the XF Real-Time ATP Rate Assay Kit (103592-100, Agilent), respectively. Finally, cell counting post the XF metabolic assay was performed using an array of Scan High-Content System (Thermo Fisher Scientific, Waltham, MA, United States) *via* DAPI staining. Mitochondrial respiratory capacity and the ATP production rate were normalized to total cell numbers and showed as pmol/min/10,000 cells.

### Blue Native Polyacrylamide Gel Electrophoresis (BN-PAGE)

Mitochondria were firstly isolated from aconitine (5 μM) treated NRVMs using a Mitochondria Isolation Kit (C1260, Applygen) according to the manufacturer’s instructions. Briefly, 5 × 10^7^ cells were harvested and resuspended in an ice-cold mito solution, followed by 40 gentle up-and-down grindings in a glass homogenizer. The cellular homogenate was transferred into a 1.5 ml tube, and mitochondria were isolated by gradient centrifugation as reported before (Zhou et al., 2013). Immediately, mitochondrial membrane proteins were homogenized in solubilization buffer before adding 6 μl 20% digitonin and centrifuged at 100,000 g for 15 min. Furthermore, 5 μl of 50% glycerol and 3 μl of 5% Coomassie blue G-250 were added into the supernatants to prevent protein aggregation in the sample gel. Subsequently, purified mitochondrial proteins were separated in 3.5 sample gel and gradient separation gel (4% acrylamide and 13% acrylamide mixture) at 100 V at 4°C. Once the dye marker migrated to the edge of separating gel, electrophoresis was performed with the current less than 15 mA and the voltage less than 500 V. When the blue running front approached 1/3 of the total running distance, the dark blue cathode buffer B was removed, and the electrophoresis was successively run using 10% cathode buffer B. At the end of electrophoresis, the gel was fixed in the solution (50% ddH_2_O, 40% methanol, and 10% acetic acid) for 30 min and washed 15 min (× 4), followed by Coomassie staining overnight. The stained gel was scanned and visualized by an automatic exposure system (ImageQuant LAS500).

### LC-MS/MS-Based Proteomics Analysis

Two separate LC-MS/MS-based proteomics analyses were performed in control and 5 μM aconitine-treated groups. In brief, the mitochondrial protein extracts were dissolved in lysis buffer, and the concentrations were determined by Bradford assay. Then lyophilized proteins from the two groups were redissolved in the solution (50 μl) containing equal ratios of urea (8 M) and DTT (20 mM) and incubated in a water bath for 4 h at 37°C. An equal volume of IAA (100 mM) was added to each group and then incubated for 1 h in dark. Next, NH_4_HCO_3_ (50 mM) buffer was added to the samples until the concentration of urea became 1 M. Subsequently, trypsin was added at a 1:50 mass ratio of trypsin: protein and digested at 37°C overnight. Finally, the digested peptides were freeze-dried and redissolved with 0.1% formic acid. The prepared sample was analyzed by a Q Exactive HF spectrometer (Thermo Fisher Scientific) coupled online with a nanoflow liquid chromatography system (Easy-nLC 1200, Thermo Fisher Scientific). All data were acquired using Xcalibur software (Thermo Fisher Scientific). After LC-MS/MS analysis, protein identification was performed by searching against UniProt databases using Mascot software (Matrix Science, London, United Kingdom).

### Statistical Analysis

All data were expressed as mean ± SD. And the statistical comparisons between groups were analyzed by one-way ANOVA followed by LSD post hoc pair-wise comparisons test. *p* < 0.05 was considered statistically significant.

## Results

### Repeated Low Dose of Aconitine Treatment Induced Undetectable Cytotoxicity on NRVMs and Promoted Myocardial Contraction

**FIGURE 1:**
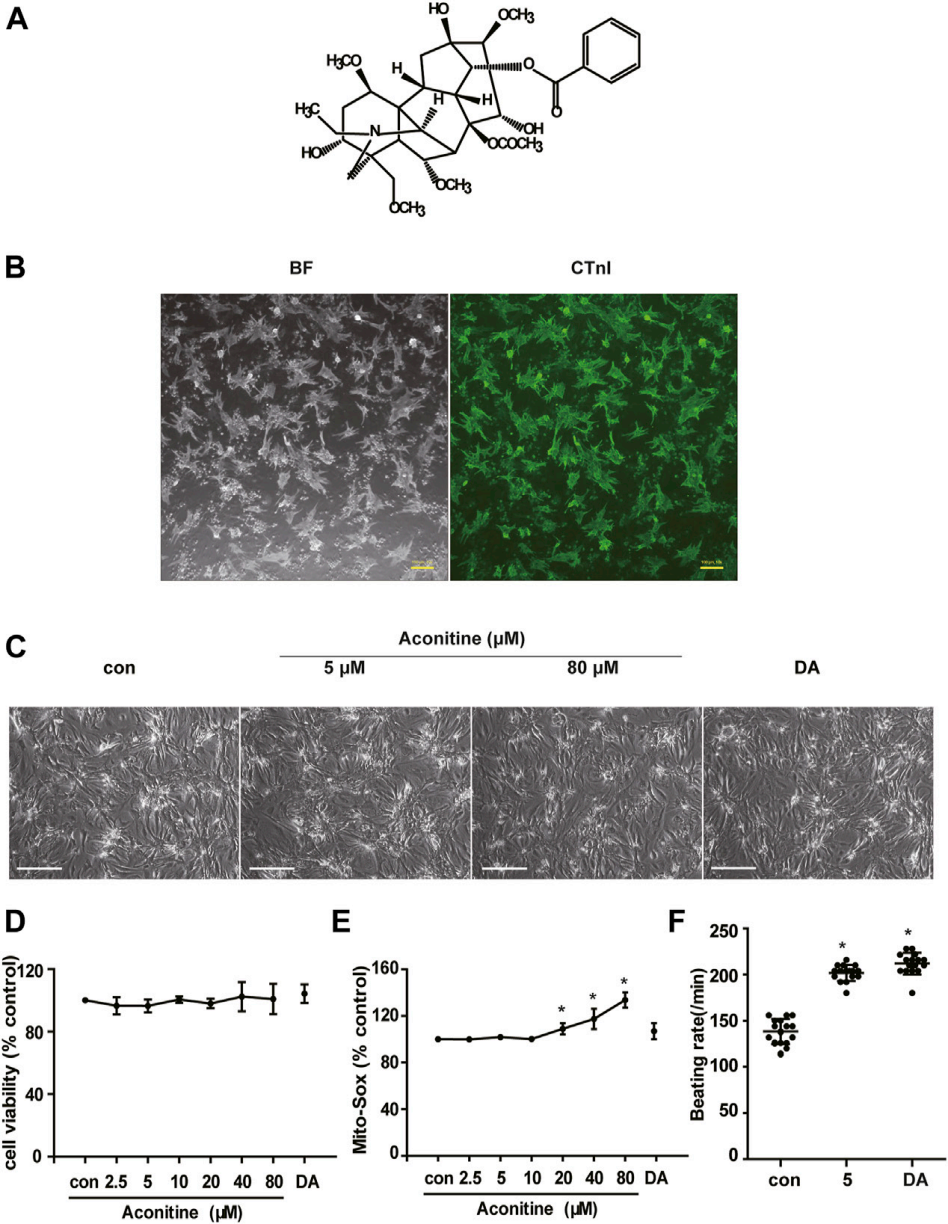
Cytotoxicity of repeated low dose of aconitine on NRVMs. **(A)** Structure formula of aconitine. **(B)** The identification and purity of NRVMs. BF represents the bright field, and the green indicates the fluorescence of CTnI. Scale bars: 100 μm. **(C)** Morphology of NRVMs after treated with different concentrations of aconitine (0, 5, and 80 μM) and DA (50 μM). Scale bars: 200 μm. **(D**,**E)** The cell viability and mitochondrial superoxide level of NRVMs treated with aconitine or DA for repeated 7 days (*n* = 3). **(F)** Effects of treatment with aconitine (5 μM) and DA (50 μM) for 7 days on the beating rates of NRVMs (*n* = 16). **** *p* < 0.05 vs. control.

To examine whether cumulative aconitine administration could cause cytotoxicity on cardiomyocytes, we repeatedly treated purified NRVMs with 0–80 μM aconitine (Figures 1A,B) for 7 days. Furthermore, DA (50 μM) was used as a positive control drug (Begieneman et al., 2016). Intriguingly, we found that repeated aconitine (0, 5, and 80 μM) and DA treatment for 7 days did not affect the morphology of the NRVMs (Figure 1C). Similarly, 0–10 μM of aconitine and DA did not exert any impact on cell viability and generated no significant enhancement in oxidative stress status (Figures 1D,E). During treatment, we observed that aconitine (5 μM) and DA could notably increase the beating rate of cardiomyocytes (Figure 1F), accompanying with less beat amplitude (shown in Supplementary Videos S1, S2). Meanwhile, we also found that aconitine (20–80 μM) did not induce significant alterations in the morphology of NRVMs, but obvious dysrhythmia, lower contractility, and even beating stop were detected in these groups. To evaluate how cardiomyocytes afford energy supply under the process of aconitine-induced cardiotonic effect, we chose to mainly explore the efficacy of aconitine and underlying mechanisms at a no-toxic-effect dose level (less than 10 µM).

### Aconitine Prompted Mitochondrial Turnover in NRVMs at a Certain Dose Range

**FIGURE 2:**
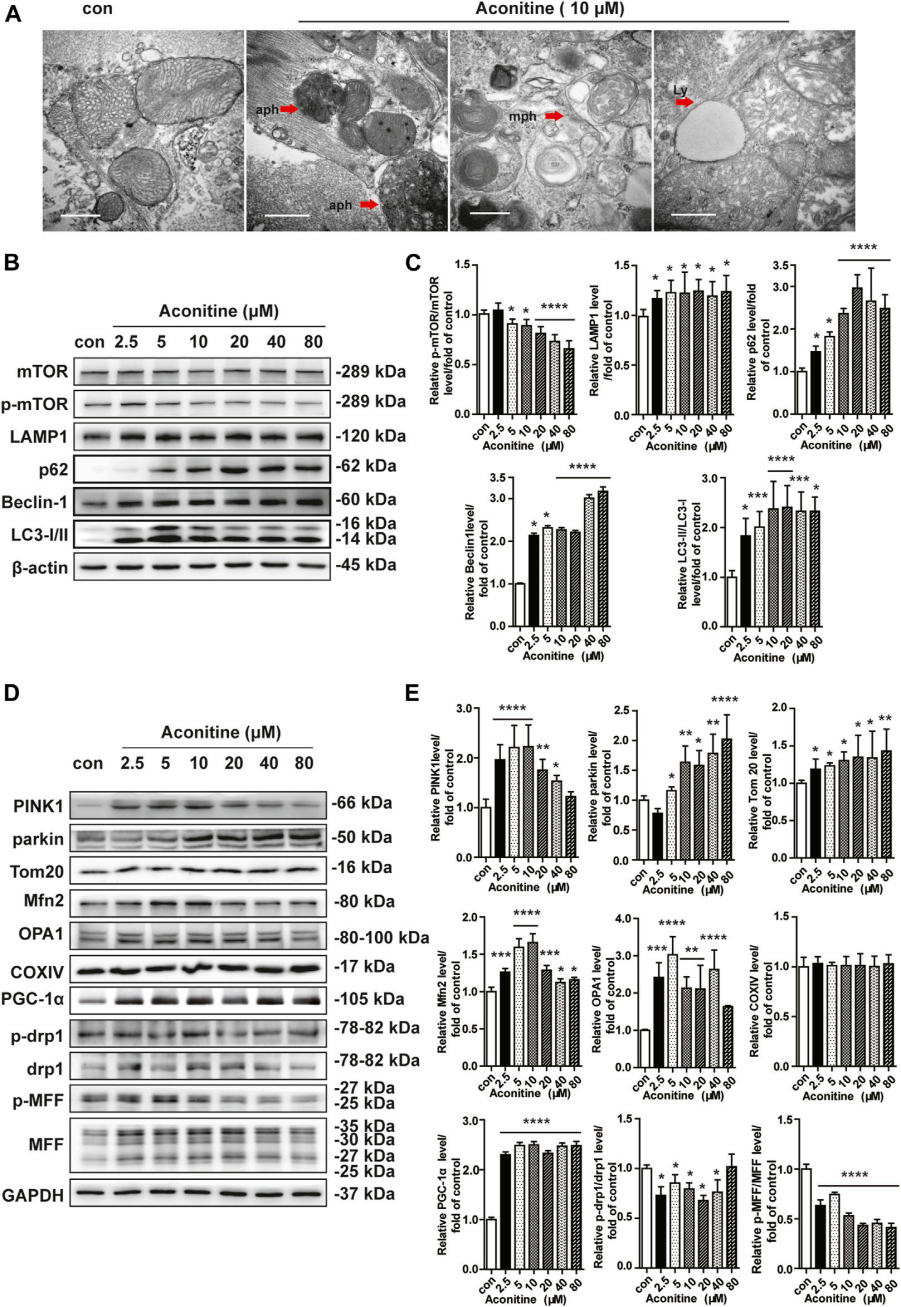
Enhanced mito-turnover in NRVMs induced by repeated treatment with aconitine. **(A)** Representative transmission electron microscopy (TEM) micrographs of NRVMs treated with aconitine (0 and 10 μM). Autophagosomes (aph), mitophagosomes (mph), and lysosome (ly). Scale bars: 1 μm. **(B–E)** Western blot and quantitative analysis of mito-turnover–related proteins. Band intensity was normalized to ß-actin or GAPDH, and presented as fold change relative to control (*n* = 3), ^*^ *p* < 0.05, ^**^ *p* < 0.01, ^***^ *p* < 0.001, ^****^ *p* < 0.0001 vs. control.

Mitochondria are highly dynamic organelles; thus, the balance of mitochondrial homeostasis plays a critical role in their function. First, we performed TEM analysis and found that aconitine enhanced the formation of autophagosomes, mitophagy, and obvious lysosomes in neonatal cardiomyocytes (Figure 2A). Furthermore, as shown in Figures 2B,C, we found that more than 5 μM of aconitine could downregulate the expression of p-mTOR while upregulating mTOR protein levels, resulting in a decrease in the ratio of p-mTOR/mTOR. Meanwhile, the expression levels of beclin1, LC3 lipidation (LC3A was converted to LC3B), and LAMP1 were significantly increased, together with the enhanced content of p62 protein, implying remarkable macroautophagy and the block of autophagic flux caused by repeated treatment with more than 5 μM of aconitine in NRVMs. Also, the proteins participating in mitophagy and biogenesis, such as Parkin, Tom20, and PGC-1α were significantly increased in a dose-dependent manner after aconitine treatment (Figures 2D,E). Aconitine-induced PINK1 and fusion-related proteins (OPA1 and Mfn2) peaked at 5 or 10 μM and began to reduce with increased dosage (Figures 2D,E). However, repeated doses of aconitine decreased the expression of mitochondrial fission-related proteins (p-Drp1 and p-MFF, Figures 2D,E). Altogether, our data suggested that aconitine might promote mito-turnover through mitophagy, mitochondrial fusion, and biogenesis at low levels (less than 10 μM).

### Effect of Repeated Aconitine Treatment on the Mitochondrial Fusion in NRVMs

**FIGURE 3:**
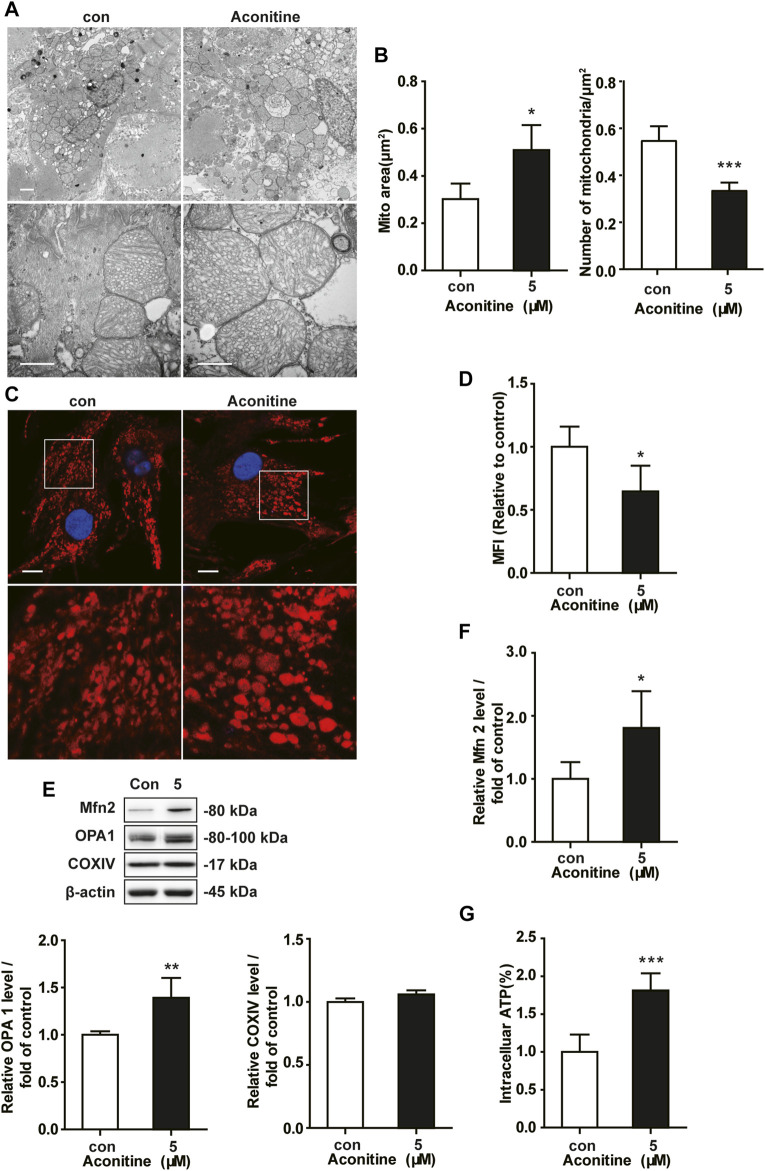
Increment of mitochondrial fusion induced by repeated low dose of aconitine treatment. **(A)** Representative TEM images of NRVMs treated with aconitine (0 and 5 μM). Scale bars: 1 μm. **(B)** The mean values of mitochondrial area (μm^2^) and the number of mitochondria per μm^2^ (*n* = 3, 100 mitochondria per group) in the two groups. **(C)** Representative confocal images of mitochondrial morphology in control or 5 μM groups. Scale bars: 10 μm. **(D)** Quantification of mitochondrial fragmentation using mitochondrial fragmentation index (MFI) (*n* = 20). **(E**, **F)** Western blot analysis of the expression of indicated proteins in NRVMs. All data were normalized to ß-actin and expressed as fold-change over control (*n* = 3). **(G)** The levels of intracellular ATP (*n* = 5). ^*^ *p* < 0.05, ^**^ *p* < 0.01, ^***^ *p* < 0.001 vs. control.

To characterize whether the morphology of mitochondria was altered by repeated 7-day low dose of aconitine (5 μM) exposure, we carried out a TEM assay and presented notable changes in mitochondrial morphologic features that mitochondria exhibited enlarged size and decreased number but no significant abnormal structure in the treated myocytes (Figure 3A). And we found that aconitine treatment resulted in an increment (from 0.3022 to 0.5090) in the mitochondrial area compared with that in the control group, which was concerned with a decrease in the number of mitochondrial areas (from 0.5232 to 0.3375) (Figure 3B). To further verify this finding, we next quantified the noncontinuous (fragment) mitochondria and calculated the MFI in aconitine-treated NRVMs using immunofluorescence. Also, mitochondria became bigger and the mitochondrial density diminished in the treated NRVMs (Figure 3C). Besides, we found that aconitine administration significantly decreased the MFI compared with that of the control group (Figure 3D), suggesting that aconitine treatment indeed caused mitochondrial fusion in NRVMs. Consistently, aconitine treatment indeed increased the expressions of Mfn2 and OPA1 but did not exert impact on the content of COX IV (Figures 3E,F). Notably, we found that repeated treatment with aconitine and DA both could upregulate cellular ATP content in cardiomyocytes (Figure 3G). Therefore, our data implied that 5 μM of aconitine treatment might improve mitochondrial function through enhanced OPA1- or Mfn2-dependent mitochondrial fusion in cardiomyocytes.

### Aconitine Increased the Mitochondrial Function by Upregulating ATP5A1 Expression in NRVMs

**FIGURE 4:**
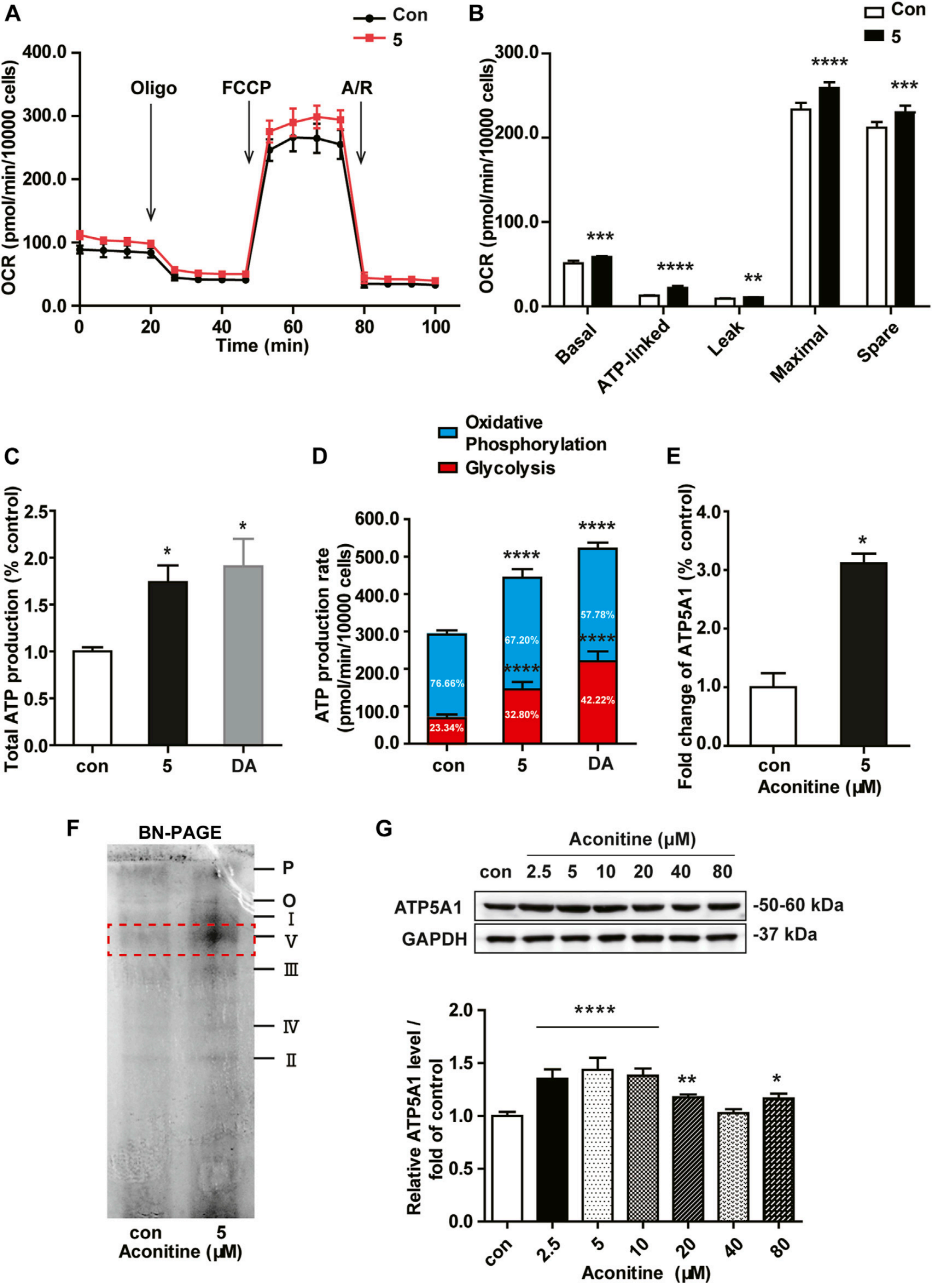
Low dose of aconitine increased mitochondrial respiration by promoting ATP5A1 in NRVMs. **(A)** Mitochondrial function of NRVMs with aconitine (0 and 5 μM) were obtained *via* the Seahorse XF Cell Mito Stress Test, and representative time course data for indicated NRVMs are shown. **(B)** Quantitive analysis of OCRs (*n* = 8). **(C)** The total ATP production. **(D)** The quantification of ATP production rate in NRVMs (*n* = 8). **(E)** Aconitine-induced fold change in ATP5A1 obtained by LC-MS/MS-based proteomic analysis (*n* = 3). **(F)** The content of various respiratory chain complexes in NRVMs treated with aconitine (0 and 5 μM) using BN-PAGE. **(G)** Western blot analysis of ATP5A1 expression in NRVMs treated with aconitine for 7 days. The data represented relative expression to that of control by normalizing to GAPDH (*n* = 3). ^*^ *p* < 0.05, ^**^ *p* < 0.01, ^***^ *p* < 0.001, ^****^ *p* < 0.0001 vs. control.

As shown in Figure 4A, OCRs in rat neonatal cardiomyocytes revealed that repeated dosing of aconitine and DA both significantly increased mitochondrial basal respiration, ATP production, maximal respiration, and spare respiratory capacity, suggesting a higher cellular respiratory function in NRVMs (Figure 4B). Also, there was no difference in proton leak in aconitine-treated cells, indicating mitochondria were not damaged at this low level. Of note, aconitine and DA induced the increase in ATP content were also evidenced by the following real-time ATP rate assay, and the fractions of ATP produced from OXPHOS in aconitine group were raised by about 1.5 times (Figures 4C,D). Next, we found that repeated 5 μM of aconitine markedly enhanced the content of ATP synthase (complex V, Figure 4F). Moreover, LC-MS/MS analyses revealed that 5 μM of aconitine induced a significantly increased expression of ATP5A1 in NRVMs (Figure 4E). Subsequently, we performed Western blotting and found repeated aconitine treatment increased the expression of ATP5A1 (Figure 4G). Together, these results suggested that repeated low dose of aconitine could enhance mitochondrial function *via* ATP5A1-mediated energy synthesis.

### Aconitine Regulated OPA1-Mediated Mitochondrial Fusion *via* Activation of AMPK Signaling in NRVMs

**FIGURE 5:**
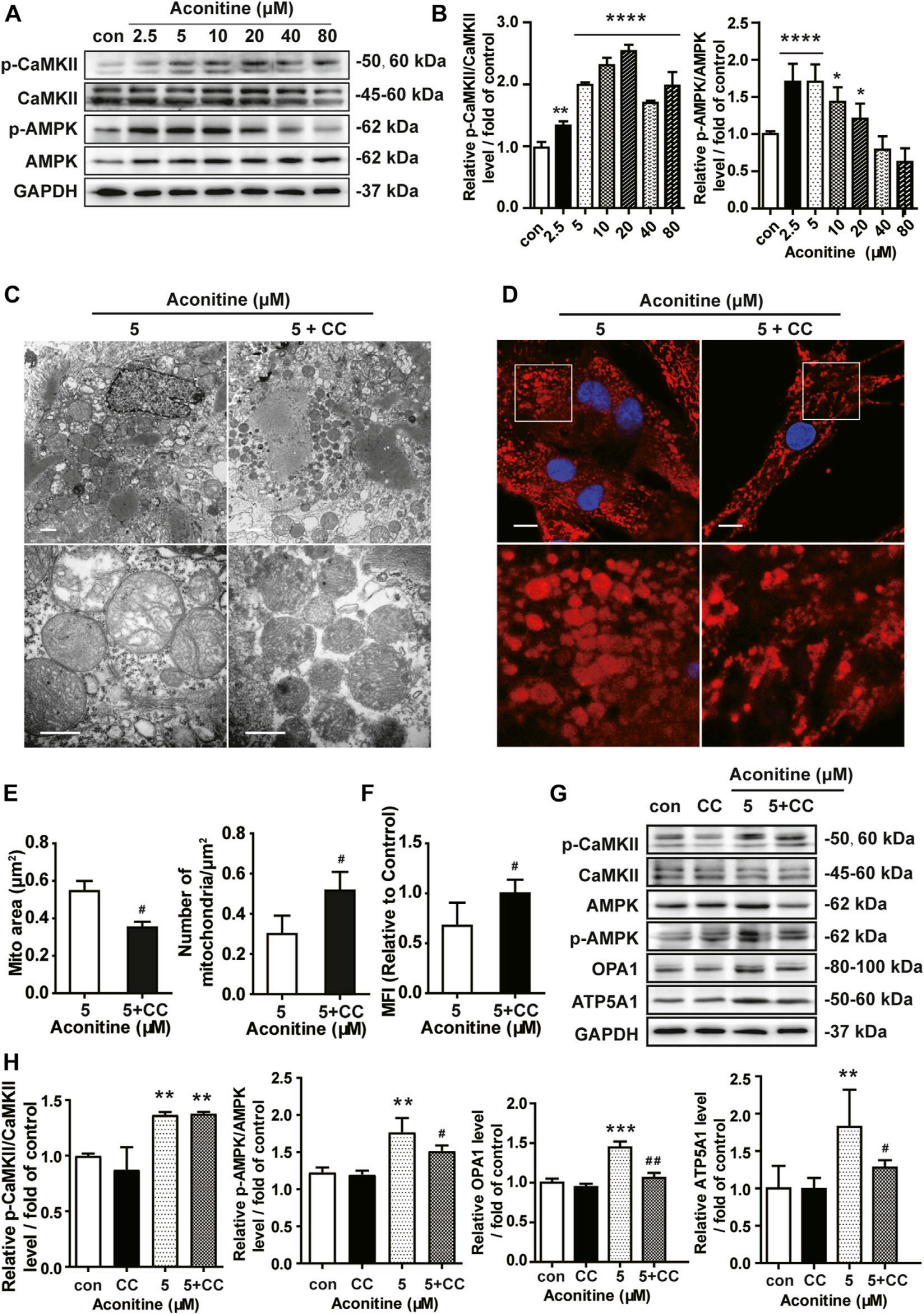
Aconitine increased OPA1-mediated mitochondrial fusion *via* activation of AMPK signaling in NRVMs. **(A**, **B)** The effects of different treatments on indicated proteins were measured by Western blot. Band intensity was normalized to GAPDH and data presented as fold change relative to control. **(C**, **D)** Representative TEM and confocal images of mitochondria in aconitine-treated NRVMs with or without compound C (CC) co-treatment. Scales represent 1 μm. **(E)** Quantification of the mitochondrial area (μm^2^) and the number of mitochondrial per μm^2^ (*n* = 3, 100 mitochondria per group). **(F)** MFI for mitochondria in the two groups (*n* = 20). **(G**, **H)** Western analysis of expression of indicated proteins in NRVMs co-treated with CC and aconitine. All data were normalized to GAPDH and expressed as fold-change over control (*n* = 3). ^*^ *p* < 0.05, ^**^ *p* < 0.01, ^***^ *p* < 0.001, ^****^ *p* < 0.0001 vs. control, ^*#*^ *p* < 0.05, ^*##*^ *p* < 0.01 vs. 5 μM.

**FIGURE 6:**
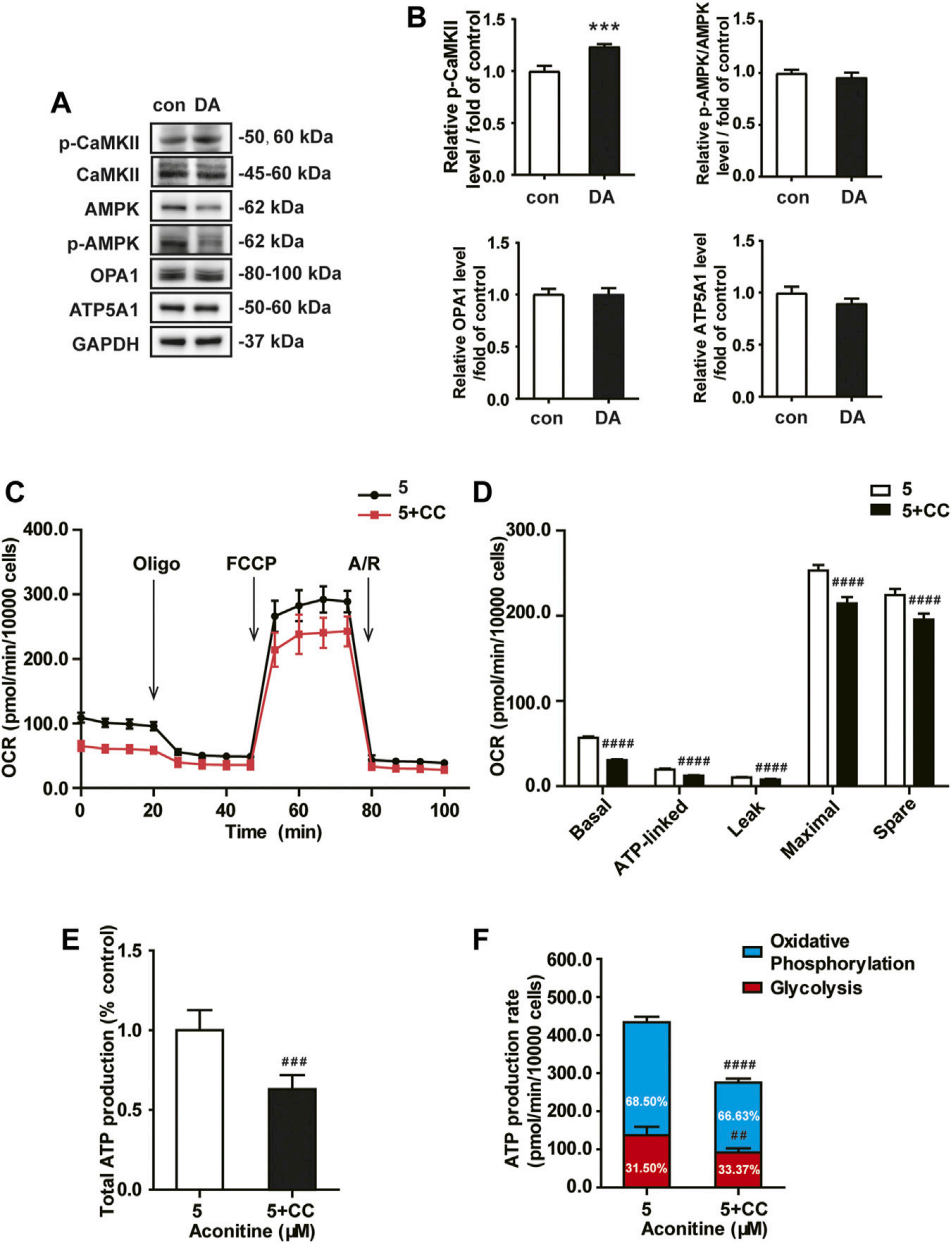
Aconitine-enhanced mitochondrial function was reversed by the AMPK inhibitor in NRVMs. **(A**, **B)** Western analysis of expression of indicated proteins in NRVMs treated with DA. All data were normalized to GAPDH and expressed as fold-change over control. **(C**, **D)** Mitochondrial respiratory function in NRVMs co-treated with CC and aconitine (*n* = 8). **(E)** The total ATP production. **(F)** The quantification of ATP production rate in NRVMs co-treated with CC and aconitine (*n* = 6). ***p < 0.001 vs. control, ^*##*^ *p* < 0.01, ^*###*^ *p* < 0.001, ^*####*^ *p* < 0.0001 vs. 5 μM.

Next, we assessed the role of the AMPK signaling in aconitine-induced mitochondrial fusion and found that after repeated 0–80 μM aconitine treatment for 7 days, the ratio of p-CaMKII/CaMKII and p-AMPK/AMPK were firstly upregulated at lower levels but then began to decline with the dose increasing in the NRVMs (Figures 5A,B). Interestingly, when we co-treated NRVMs with aconitine and CC, aconitine-induced increasing in the contents of phosphor-AMPK and OPA1 were notably reversed, while there was no inhibition on p-CaMKII (Figures 5G,H). As expected, our TEM and confocal results also demonstrated that the alternations in mitochondrial morphology and the MFI caused by 5 μM of aconitine were both attenuated by CC pretreatment (Figures 5C–F). Overall, these results illustrated that phosphorylation of the AMPK signaling pathway, activated by aconitine, could contribute to OPA1-mediated mitochondrial fusion. Interestingly, DA induced an increase in the ratio of p-CaMKII/CaMKII without changing the ratio of p-AMPK/AMPK and the expressions of OPA1 and ATP5A1, suggesting the AMPK–OPA1–ATP5A1 signaling pathway did not involve in DA-induced cardiotonic effect on myocytes (Figures 6A,B).

### Effect of AMPK Inhibitor on Aconitine-Enhanced Mitochondrial Function in NRVMs

Meanwhile, inhibiting AMPK phosphorylation *via* CC caused an evident decline in ATP5A1 content in NRVMs (Figures 5G,H), implying that AMPK/OPA1 might be an upstream signaling for aconitine-induced increasing in ATP5A1. The OCRs value associated with mitochondrial function also returned to a similar level compared to that of the control group (Figures 6C,D). And as shown in Figure 6E, AMPK inhibitor could reverse the increase in cellular ATP content induced by aconitine but could not restore the ratio of OXPHOS to glycolysis in NRVMs (Figure 6F). These results demonstrated that repeated aconitine treatment at a low level (5 μM) was responsible for the enhancement of mitochondrial function in NRVMs, and the AMPK signaling pathway could play an important role during this process. Taken together, our data suggested that repeated aconitine (5 μM) treatment facilitated OPA1-mediated mitochondrial fusion and the following mitochondrial function, during which AMPK–OPA1–ATP5A1 signaling could be involved in and play a crucial role in low dose of aconitine-induced cardiotonic effect.

## Discussion

In the current study, we first found that repeated treatment with low doses (0–10 μM) of aconitine for 7 days could accelerate the mitochondrial turnover characterized by activating mitophagy, mitochondrial fusion, and biogenesis in cardiomyocytes, all of which eventually contributed to the enhancement of mitochondrial function and ATP production, and induced positive intronic effect. Significantly, the activation of the AMPK–OPA1–ATP5A1 signaling pathway played a crucial role in this process. All of these results may provide a possible mechanism involved in aconitine-induced regulation of energy metabolism, which is necessary to the maintenance of cardiotonic effect induced by medicinal plants containing aconitine.

Since the excellent pharmacological effect, herbs containing aconitine are widely used to treat cardiovascular diseases, such as heart failure, in China and other East Asian countries (Wu et al., 2019). But in the past decades, the studies on aconitine mostly focused on single or short-term administration at extremely high doses and overemphasized the toxicity of these ancient herbs (Jacobs and Haydock, 2019; Peng et al., 2020). However, in disagreement with previous reports (Gao et al., 2018; Ji et al., 2019), we did not find any detectable cytotoxic effects induced by repeated aconitine treatment (0–10 μM). Consistently, this study found that 5 μM of aconitine indeed generated a positive inotropic effect on cardiomyocytes characterized by the increased beating rate (Tak et al., 2016; Sun et al., 2018). However, matched energy metabolism supporting such high frequency beating and underlying mechanism remain poorly understood.

Mitochondria, as the main sites of energy metabolism, provide more than 90% of ATP supply through oxidative phosphorylation (Van der Bliek et al., 2017). Also, mitochondria are highly dynamic organelles and always being the homeostasis or named mitochondrial turnover, which consists of biogenesis, mitophagy, and fission/fusion and is critical for mitochondrial function (Liesa et al., 2009; Westermann, 2010). Generally, mitochondrial fission contributes to the redistribution of mitochondria, mitophagy removes damaged or dysfunctional mitochondria, and biogenesis and fusion promote a healthy mitochondrial network, and all of these processes are precisely regulated (Dorn, 2019). In our study, we firstly found that more than 10 μM of aconitine mainly induced autophagy and blockage of autophagic flux, evidenced by a dose-dependent increase in autophagy-related proteins (LC3B, Beclin1, and p62, and LAMP1) and autophagic vacuoles in myocytes. However, in the groups treated with lower doses of aconitine, the enhanced protein content of Parkin and PINK1 and degraded mitochondria in autophagosome or nearby lysosome suggested the PINK1–Parkin system-mediated mitophagy was activated in these groups. Besides, the expression of PGC1α, Mfn2, and OPA1, three important proteins controlling mitochondrial biogenesis and fusion, was increased in lower-dose groups, while aconitine did not cause any changes in the content of fission-related proteins (p-Drp1, Drp1, p-MFF, and MFF). Therefore, we deemed that repeated treatment with low doses of aconitine could promote mitochondrial turnover, particularly mitochondrial fusion.

Moreover, our results showed significant increases in the function of respiratory chain and ATP content in aconitine-treated NRVMs, and compared to DA, 5 μM of aconitine generated the same positive inotropic effect but with less glycolysis, both implying that repeated aconitine caused obvious alterations in mitochondrial energy metabolism. Considering no changes in the content of mitochondria, confirmed by cellular COX IV expression, aconitine-induced enhancement in energy supply might mostly result from alterations in mitochondrial function but not from mitochondrial quantity.

Generally, mitochondrial function is intimately connected with many complexes involved in the tricarboxylic acid cycle and OXPHOS and located in mitochondrial cristae (Cogliati et al., 2013). In other words, the alternations in cristae are related to the stability of respiratory supercomplexes and also affect the efficiency of mitochondrial respiratory function (Enríquez, 2016). OPA1 mainly controls mitochondrial fusion at the inner mitochondrial membrane and has been reported to participate in mitochondrial cristae remodeling by cross-talking with the mitochondrial contact site and cristae organizing system (MICOS) data not shown, a series of key factors responsible for the regulation of cristae morphology (Muñoz-Gómez et al., 2015; Quintana-Cabrera et al., 2018). Subsequently, we performed mitochondrial proteomic analysis and found that the expression of MICOS was remarkably enhanced after 7 days’ repeated treatment with 5 μM of aconitine, accompanied with obvious changes in the morphology of mitochondrial cristae in aconitine-treated myocytes, indicating that aconitine treatment could cause reshaping in mitochondrial cristae during OPA1-mediated fusion. Furthermore, OPA1 could also promote ATP synthase oligomerization and preserve mitochondrial function in cardiac tissue (Wai et al., 2015). Remarkable increases in the content of complex V (ATP synthase), ATP5A1, and ADP/ATP translocase 1 (ANT) data not shown also indicated that OPA1-mediated mitochondrial fusion might play an indispensable role in aconitine-induced remodeling in mitochondrial cristae and respiratory chain complexes. Altogether, aconitine-induced OPA1-mediated mitochondrial fusion and following elevated mitochondrial function could be conducive to meeting the increased energy demands of the fast-beating myocytes.

**FIGURE 7:**
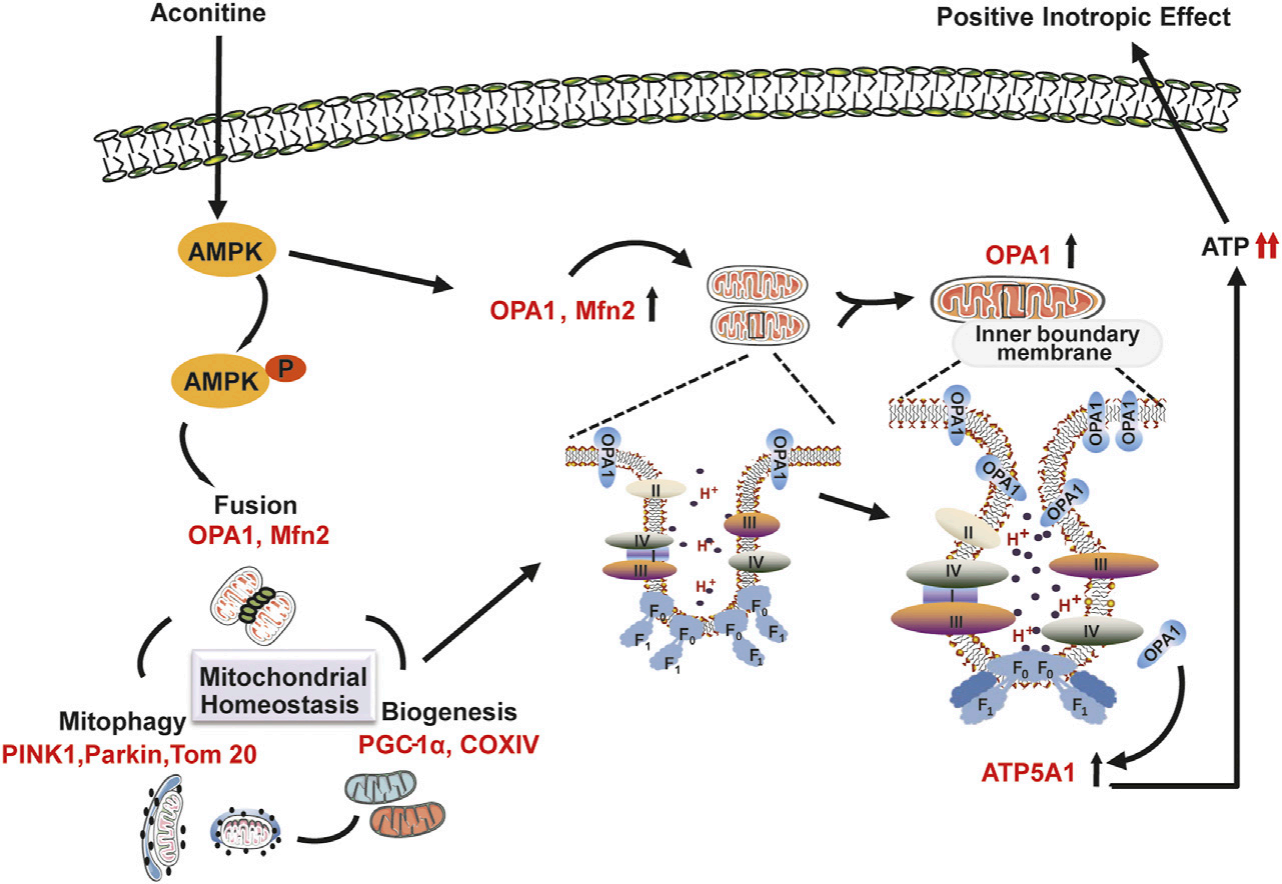
Schematic representation of the possible molecular model of action underlying aconitine-induced cardiotonic effect–related energy metabolism.

Many studies have confirmed that AMPK played a crucial role in bioenergetic metabolism, for it could quickly sense the changes in cellular ATP levels (Herzig and Shaw, 2018). Usually, AMPK could be phosphorylated by CaMKII, a Ca^2+^-activated protein kinase to timely regulate mitochondrial homeostasis (Hardie, 2011). Recently, AMPK signaling had been reported to ameliorate drug-induced hepatocyte injury *via* the enhancement of OPA1-related mitochondrial fusion (Ha et al., 2017), possibly for AMPK could inhibit the expression of mitochondrial ATP synthase β-F1 inhibitor protein, which depressed the activity of ATP synthase, resulting in the activity of oxidative respiratory chain and enhanced the ATP production (Vazquez-Martin et al., 2013; Zhang and Ma, 2018). Previous articles had reported that aconitine directly interacted with L-type Ca^2+^ channel on myocardiocytes membrane and increased the containing of cytosolic Ca^2+^ (Zhou et al., 2017) triggering downstream CaMKII–AMPK signaling (Raney and Turcotte, 2008). Consistent with the previous studies, our study observed the activation of CaMKII–AMPK signaling in aconitine-treated NRVMs (Supplementary Figure S1). And inhibiting the phosphorylated activation of AMPK abolished aconitine-increased OPA1 expression mitochondrial fusion, ATP5A1 content, mitochondrial respiratory function, and ATP production, revealing that the AMPK was indeed involved in aconitine-induced elevation in mitochondrial function and energy metabolism by regulating the expression of OPA1 and ATP5A1 in NRVMs after repeated aconitine administration. As shown in Figure 7, we also presented a possible molecular model of action of energy metabolism involved in aconitine-induced cardiotonic effect.

## Limitation

Several limitations existing in our study need to be acknowledged. First, all results acquired from *in vitro* experiments, the effect of low doses of aconitine on the cardiomyocytes should be further validated in animal experiments. Second, we only explored the mechanism underlying the cardiotonic effect induced by aconitine using normal myocardial cells, leading to limited generalizability of cardiac pharmacological effects of aconitine to the disease model. Furthermore, aconitine-induced toxicity is still the chief issue that prevents its wider use and the interpretation of therapeutic potential, and we also found the overlap between dose margin of this efficacy and that of triggering arrhythmia. Optimal therapeutic concentration of aconitine is up for a much wider scrutiny and discussion.

## Conclusions

In conclusion, we found that repeated administration of aconitine could accelerate mitochondrial turnover through mitophagy, mitochondrial biogenesis, and fusion and promote reshaping of mitochondrial cristae and ATP synthase, both of which contributed to aconitine-induced energy metabolism and provide sufficient ATP for the fast-beating myocytes. Furthermore, we first identified that AMPK–OPA1–ATP5A1 signaling pathway played an important role in this process and could be one of the crucial pharmacological mechanisms underlying aconitine-induced cardiotonic effect–related energy metabolism.

## Data Availability

The original contributions presented in the study are included in the article/Supplementary Material, and further inquiries can be directed to the corresponding author.

## Abbreviations

AMPK, AMP-activated protein kinase; ATP5A1, ATP synthase α-subunit; CC, compound C; DA, dopamine; NRVMs, neonatal rat ventricular myocytes; OPA1, optic atrophy 1.
