# Supplementary material for: Repeated Aconitine Treatment Induced the Remodeling of Mitochondrial Function via AMPK–OPA1–ATP5A1 Pathway
Source: Front Pharmacol. 2021 Jun 10;12:646121. doi: 10.3389/fphar.2021.646121 (PMC8224173; doi:10.3389/fphar.2021.646121)
Supplement: Supplementary file 1 [file Table1.DOCX]

**Supplementary Materials**

**Table S1 Information for all indicated antibodies used in IF, WB assay.**

|  | **Name** | **Company** | **Dilution rate** |
| --- | --- | --- | --- |
| 1 | CTnI(24/26KDa) | ab47003, Abcam, USA | 1:400 |
| 2 | Anti-Rb IgG | ab6721, Abcam, USA | 1:3000 |
|  | Anti-Ms IgG | ab6789, Abcam, USA | 1:3000 |
| 3 | GAPDH(36KDa) | ab181602, Abcam, USA | 1:3000 |
| 4 | β-actin(45KDa) | 4970S, Cell Signaling Technology, USA | 1:1000 |
| 5 | p-AMPK (62KDa) | ab133448, Abcam, USA | 1:1000 |
| 6 | AMPK (62KDa) | 5831S/5832S, Cell Signaling Technology, USA | 1:1000 |
| 7 | OPA1 (80-100KDa) | 80471S, Cell Signaling Technology, USA | 1:1000 |
| 8 | PGC-1α(105KDa) | Ab54481, Abcam, USA | 1:1000 |
| 9 | ATP5A1 (50-60KDa) | 14676-1-AP, Proteintech, USA | 1:1000 |
| 10 | Beclin1 (60 KDa) | 3495S, Cell Signaling Technology, USA | 1:1000 |
| 11 | LAMP1 (120 KDa) | ab24170, Abcam, USA | 1:2000 |
| 12 | LC3B (14,16 KDa) | 3868S, Cell Signaling Technology, USA | 1:3000 |
| 13 | p-MFF (25, 27, 30,35KDa) | 84580S, Cell Signaling Technology, USA | 1:1000 |
| 14 | MFF(25, 27KDa) | 84580S, Cell Signaling Technology, USA | 1:1000 |
| 15 | p-Drp1(78-82KDa) | 4494S, Cell Signaling Technology, USA | 1:1000 |
| 16 | Drp1(78-82KDa) | 5931S, Cell Signaling Technology, USA | 1:1000 |
| 17 | P62 (62 KDa) | 5114S, Cell Signaling Technology, USA | 1:1000 |
| 18 | Mfn2 (80 KDa) | 11925S, Cell Signaling Technology, USA | 1:1000 |
| 19 | PINK1 (66 KDa) | ab23707, Abcam, USA | 1:500 |
| 20 | Parkin (50 KDa) | 4211S, Cell Signaling Technology, USA | 1:1000 |
| 21 | Tom20 (16 KDa) | 42406S, Cell Signaling Technology, USA | 1:1000 |
| 22 | COX IV (17KDa) | 4850S, Cell Signaling Technology, USA | 1:1000 |
| 23 | p-mTOR (289 KDa) | 2971S/5536T, Cell Signaling Technology, USA | 1:1000 |
| 24 | mTOR (289 KDa) | 2983S, Cell Signaling Technology, USA | 1:1000 |
|  | Phospho-CaMKII (50, 60KDa) | 12716S, Cell Signaling Technology, USA | 1:1000 |
| 25 | CaMKII (45-60KDa) | 137301-1-AP, Proteintech, USA | 1:1000 |
