# Supplementary figures and images for: Repeated Aconitine Treatment Induced the Remodeling of Mitochondrial Function via AMPK–OPA1–ATP5A1 Pathway
Source: Front Pharmacol. 2021 Jun 10;12:646121. doi: 10.3389/fphar.2021.646121 (PMC8224173; doi:10.3389/fphar.2021.646121)

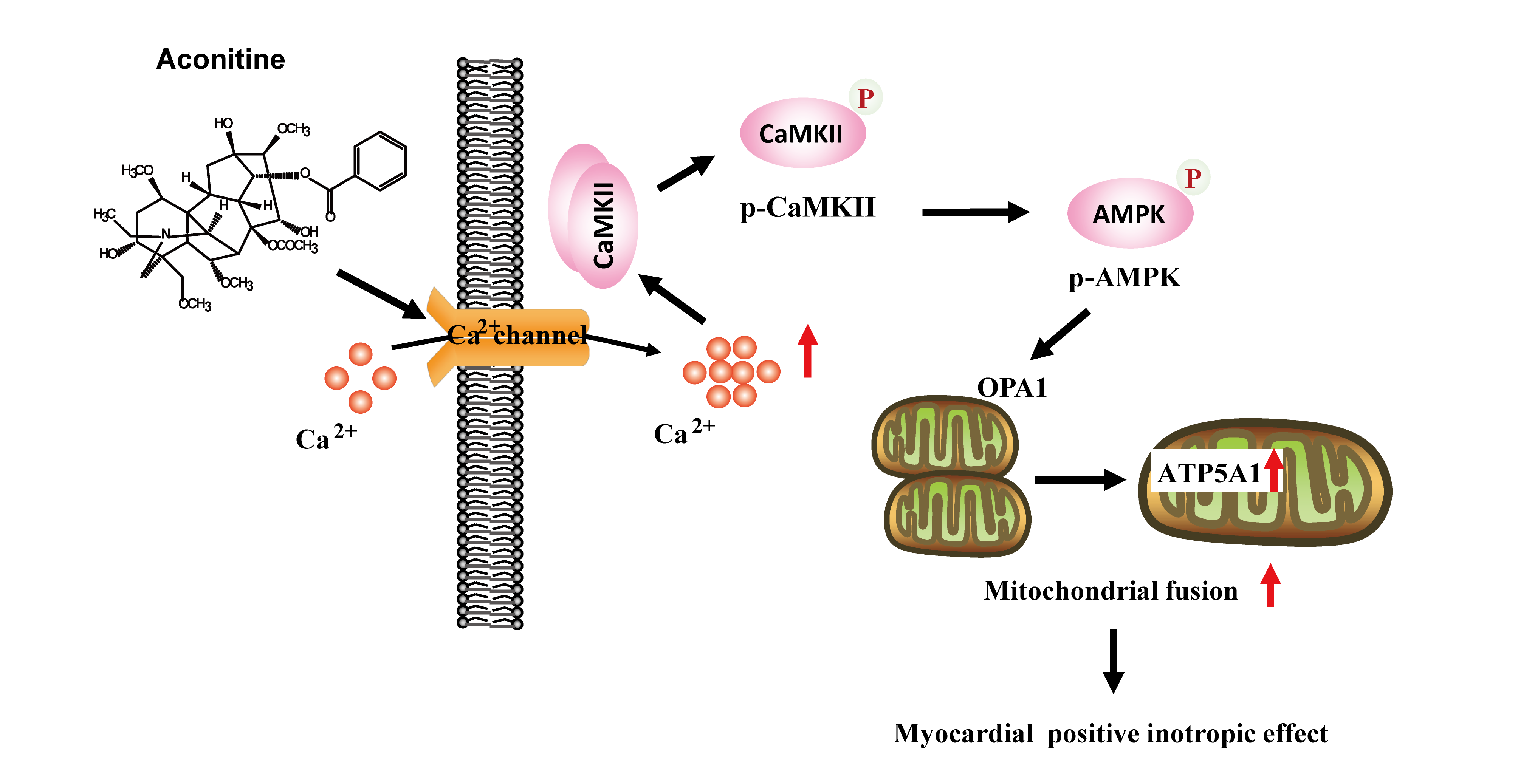

Supplement: Supplementary file 2 [file Image1.TIF]
